# Influence of Features on Accuracy of Anomaly Detection for an Energy Trading System

**DOI:** 10.3390/s21124237

**Published:** 2021-06-21

**Authors:** Hoon Ko, Kwangcheol Rim, Isabel Praça

**Affiliations:** 1 Instituto Superior de Engenharia do Porto, Instituto Politecnico do Porto, R. Dr. Antonio Bernardino de Almeida, 431, 4249-015 Porto, Portugal; hko@isep.ipp.pt; 2 College of Basic & General Education, Chosun University, 309 Pilmundae-ro, Dong-Gu, Gwangju 61452, Korea; rim1201@chosun.ac.kr

**Keywords:** anomaly signal, anomaly detection, feature analysis, cyber-attack

## Abstract

The biggest problem with conventional anomaly signal detection using features was that it was difficult to use it in real time and it requires processing of network signals. Furthermore, analyzing network signals in real-time required vast amounts of processing for each signal, as each protocol contained various pieces of information. This paper suggests anomaly detection by analyzing the relationship among each feature to the anomaly detection model. The model analyzes the anomaly of network signals based on anomaly feature detection. The selected feature for anomaly detection does not require constant network signal updates and real-time processing of these signals. When the selected features are found in the received signal, the signal is registered as a potential anomaly signal and is then steadily monitored until it is determined as either an anomaly or normal signal. In terms of the results, it determined the anomaly with 99.7% (0.997) accuracy in *f(4)(S0)* and in case *f(4)(REJ)* received 11,233 signals with a normal or 171anomaly judgment accuracy of 98.7% (0.987).

## 1. Introduction

Many servers are potential victims of cyber-attack. An energy trade system, for instance, is prone to cyber attack. In [1], transaction of energy is carried out within a transparent network that consists of smart factories, solar systems, home solar systems, wearable devices, smart IoT devices, smart vehicles and personal offices in smart cities. Relevant parties trade energy on the energy network by sending each other messages such as *reply* or *request*. Although the transparency of the network ensures the security of the parties from direct hacking, the energy network can be monitored and network data collected by an attacker can use it to prey on other parties anonymously [2]. In order to reveal the perpetrator, the system has to detect abnormal signals out of the energy trading signals and alert security. This paper suggests anomaly detection by analyzing the relationship between each feature to the anomaly detection model. The model analyzes the anomaly of network signals based on abnormal feature detection. The selected feature for anomaly detection does not require constant network signal updates and real-time processing of these signals. When the selected features are found in the received signal, the signal is registered as a potential anomaly signal, and is then steadily monitored until it is determined as either an anomaly or normal signal. The function as a surveillance for anomaly features in network signals. It detects and tracks potential abnormal signals until the nature of the signal is determined. This study attempts to analyze the features of network signals and compare the results of feature analysis for anomaly detection. Finally, it can be utilized as a precautionary measure for anomaly detection, and is expected to secure the safety of energy trading systems using energy networks. Works related to the security issue are introduced in Section 2, and this followed by the definition of the proposed ADM in Section 3. Section 4 defines the features used by the model. This study focuses on the *service3(f3)* and *flag(f4)*, and the varied performance levels of models trained by these features are given in Section 5. The paper is then concluded in Section 6.

## 2. Related Works

### 2.1. Anomaly Detection

**Figure 1:**
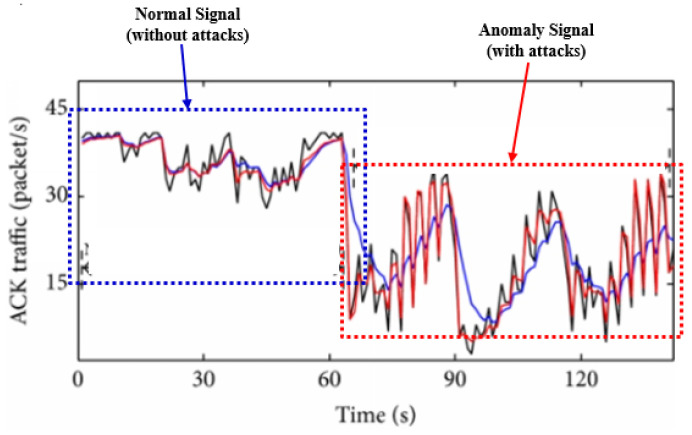
Normal Signal (NS) vs. Anomaly Signal (AS).

In [3], the research analyzes ML (Machine Learning) applications across IoT data processing and management functions, study directions, and difficulties. The authors worked on how to use ML in numerous IoT contexts which are the attempts to bring headlines to the forefront. This paper included the applications of ML across management functions as well as IoT data processing. In [4], the study offers a scientific classification of some dangers of the existing IoT security, and provides a guide for novel and energizing research difficulties in applying ML and SDN (Software-Defined Network) ideas to address IoT security concerns. In [5], the authors reviewed the applications of ML in cybersecurity. There has also been a debate on the risks of utilizing cyber attacks as training and testing data for classification. In [6], the authors surveyed the overviews that have used ML and DL (Deep Learning) in the related research areas such as networking, communications, and lossy environment. The primary objective of this survey study is to distinguish potential topics and challenging tasks for using different deep learning and machine learning algorithms. In addition the surveys all overview the several works that have used the ML/DL techniques which include networks, communications and so on. The main object of those studies is to distinguish the potential topics and challenging tasks for using the ML and DL algorithm. In [7], the authors study different machine learning and deep learning algorithms used to develop network intrusion detection and strategies used to define cybersecurity and intrusion detection accuracy in addition to existing blockchain technology applications. In [2], the authors suggested an intrusion detection by smart meters for cybersecurity. The strategy attempts to detect intrusions by monitoring computer systems, such as memory, storage, CPU resources, and all network traffic, with a smart meter. However, the system is impractical as it has to check for all resources. In [8], the authors analyzed some anomaly detection for cybersecurity based on CNN (Convolution Neural Networks) as a survey. CNN is utilized for its ability to process an input-value with multiple dimensions. Almost all solutions which have been studied usually have gathered and classified the input data and perform a pre-processing.So, if the pre-processing is wrong, the result for anomaly detection would be not good. To solve this, this study in [8] suggested the unified cross framework which simulates end-to-end anomaly detection mechanisms. Authors in [9] applied detailed research methods to the safety problems and dependable tactics from a data-driven viewpoint, when learning and evaluating or inferring machine learning. The authors emphasized the drifting of data distribution and the leakages of the sensitive information issues in predictable ML algorithms. In [10], the authors studied anomaly detection in machine learning of network analysis of intrusion detection and define a brief walkthrough summary of the ML and DL techniques. In [11], anomaly signals are detected from normal signals. The statistics results of traffic based on the AEWMA (Adaptive Exponentially Weighted Moving Average) algorithm and the EWMA (Exponentially Weighted Moving Average) algorithm are shown in Figure 1.

Figure 1 shows what AEWMA (Adaptive Exponentially Weighted Moving Average) algorithm and the EWMA (Exponentially Weighted Moving Average) algorithm can effectively smooth the slight fluctuation of the traffic when the traffic which has no attacks is normal. The two curves’ corresponding statistical values almost coincide. However, when the traffic under attacks is anomaly, the EWMA algorithm smooths the large fluctuation too, while the AEWMA algorithm can retain the anomaly characteristics of the sample value. The corresponding statistical values of the two curves are quite different. Therefore, the AEWMA algorithm is more suitable than the EWMA algorithm for DDoS attack detection based on the anomaly characteristics of traffic.

### 2.2. Energy Network for an Energy Trade Market

**Figure 2:**
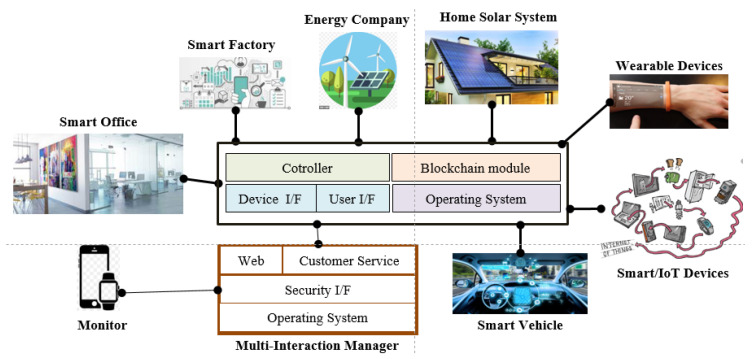
Energy Network for an Energy Trade Market.

Figure 2 shows the structure of an energy network [1] that we studied as an energy trade system which consists of a consumer, a producer, a module of blockchain , and a multi-interaction management agent. This model contains a producer and a consumer who are a representation of an interconnected user. The prosumer alternates between two roles according to what users need. The users will be granted roles only after authentication by the trusted server. Furthermore, the users will be linked to one another in a grid, and they will share the energy. With the use of the shared information, the users that want to buy request the amount, and the seller receives these purchase requests. The model processes with a bi-direction structure to sell and to produce the energy from a smart factory, an energy company, a home solar system, a wearable device/IoT device, a smart vehicle and a personal office and so on. There is also a multi-interaction manager agent (MiM agent) and the MiM agent functions as a manager of the energy trade in the energy network. The energy network consists of a controller, device I/F (Interface), user I/F, blockchain module and operation system. In Figure 2, the energy company only produces energy. The home solar system can be either a consumer or a producer. A personal office, a smart factory, wearable devices, smart IoT devices and smart vehicles are customers. Generally all participants contact an MiM agent to monitor the current information. If one of the customers wishes to buy energy, the customer contacts the MiM agent by sending a ‘request message’. If a producer wants to sell energy, the producer sends a ‘share message’. The ‘share message’ contains (available energy and cost). Consumers have the option of choosing from these offers.

## 3. Anomaly Detection Model (ADM)

### 3.1. The Collection of Network Signals

**Figure 3:**
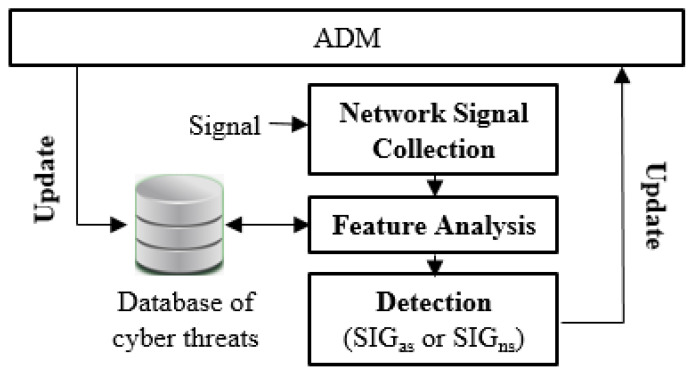
ADM (Anomaly Detection Model).

Figure 3 depicts energy generators such as the solar system and the home solar system sending a SHARE message. Depending on supply levels, at times the home solar system can sell surplus energy and vice versa [12]). To buy the energy, buyers send a (request message) to acquire the (available cost, amount available) from the ETM (Energy Trade Model). The buyer receives information and the trade is proceeded in the ETM [1] on receiving a reply message from the ETM.

**Figure 4:**
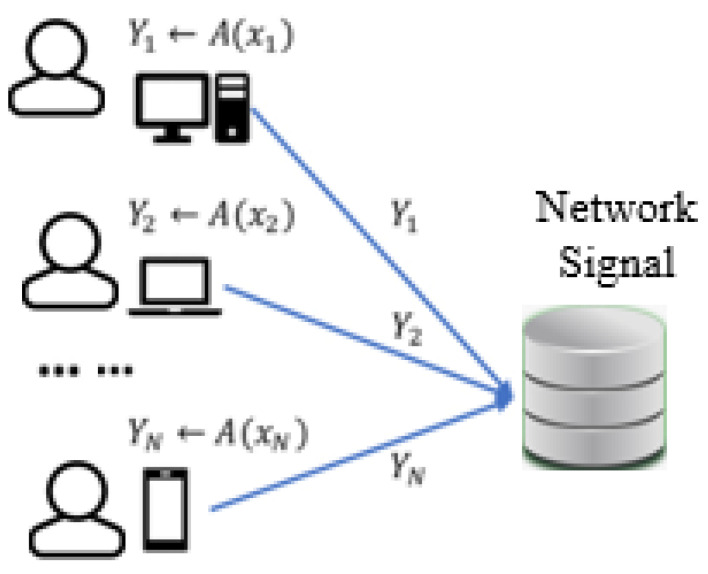
Collection of the Network Signal.

The ADM (Anomaly Detection Model) consists of the next steps: Network Signal Collection, Feature Analysis, Detection and Update (Figure 3). It runs a network signal collection as the first step. In the feature analysis step, it processes by analyzing the relationship of features. Through the feature analysis step, it decides the anomaly signal and next it updates the anomaly signals with SIG${}_{as}$ or SIG${}_{ns}$. SIG${}_{as}$ is an Anomaly Signal(as) and SIG${}_{ns}$ is a Normal Signal(ns). At the network signal collection stage depicted in Figure 4, it runs a collection algorithm ADM to encode xi from the user’s device to the data collector and it collects the Yi ← A(Xi) as output. The network signals including the normal signal and anomaly signal are collected in an array type, and they are stored as a DB (Figure 3). It is collected from each user which is proceeded by ADM [13]. *M* is a mean, *E* is an exponent, *e* is an Euler number and *A* is an array.

As the first step, it runs a collection algorithm *ADM* to encode $x_{i}$ from the user’s device to the data collector and the collected output($Y_{i}$) is next $Y_{i}=A\left(X_{i}\right)$ as the output. It needs to guarantee a type of plausible deniability, no matter what the output is collected from each user, and it would be approximately equal as it is likely to have come from more a specific value *x* as any other $x^{{}^{\prime }}$. The *ADM* follows next,

$$Y_{i}=ADM\left(X_{i}\right)=\left\{\begin{array}{c} 1,withprobability=\frac{1}{e^{E}+1}+\frac{xi}{M}\cdot \frac{e^{E}-1}{e^{E}+1}\\ 0,otherwise. \end{array}\right.$$

### 3.2. Analysis of Network Signals

Network signals are raw signals before a classification. These signals can be used as input in the model periodically or non-periodically (Figure 4). In the first step, all network signals are received disorderly. To apply ML or/and DL, it has to perform the pre-processing of the raw data because all data are received randomly or irregularly. It means that there are no rules in the flowing network signals. As we can see the data flow in Figure 1, there is a normal signal and an anomaly signal in the physical system in a time series, *s(t) = s(t${}_{0}$ + n$\tau {}_{s}$) = s(n)*, which is sampled at intervals of $\tau {}_{s}$ and initiated at *t${}_{0}$* [14]. In addition, they show up through the initial pre-processing. Furthermore, before the decision of analysis, we have no idea which the normal is or which the anomaly signal is.To analyze, the algorithm needs the data after pre-processing, but the flow of the data looks random as in a chaotic system [15]. We also surveyed the existing research, but they are not suitable for real-time. To detect the anomaly signal in the network, it should be real-time. The dataset named*KDDCup* was produced after computation. It means the dataset is not for real-time. Based on real-time, we have to select the base feature. The base feature has some qualifications which should not emerge from the computation. That feature should be captured from raw data and is simple in the first step.

## 4. Analysis

### 4.1. Feature Analysis

To simulate our suggestion, we used WEKA (ver 3.8.5) with the *KDDTrain dataset* [16]. To do this, we set ‘Discretize’ in the Preprocess and set ‘J48’ in the classifer. Moreover, we set the dataset to 80% for training and 20% for testing. The *KDDTrain dataset* is a dataset composed of 17 features from KDDCup, introduced in [16]. In the study, the selected 15 features were defined according to their respective creation and calculation methods. For example, *count(f23)* is defined by calculating the sum of connections to the same destination, or by analyzing the captured protocol without the calculation of items, such as *service(f3)* and *flag(f4)* (Table 1). As defined in Table 1, both *service(f3)* and *flag(f4)* have features defined by mathematical calculations. Table 2 shows the results of accuracy according to feature. The accuracy score of analyzing the class of *service(f3)* was 72.564%, and therefore it was considered meaningless to analyze because all network services are based on *service(f3)* including attack signals. On the other hand, the experiment’s results with *flag(f4)* all returned 99.120% in accuracy. This is because *service(f3)* is composed of applications, instructions, or protocols in a network, resulting in more than 50 classes. Since both normal signals and attack signals are normally used, analysis of *service(f3)* was concluded to be meaningless. Instead the study focuses on analyzing the relationship between classes of *flag(f4)*, the only feature that is not a feature calculated by complex calculations. Each class defined in *flag(f4)* is defined in Table 3. In addition, the descriptions of all 42 features are also provided.

### 4.2. Correlation of Each Feature

**Figure 5:**
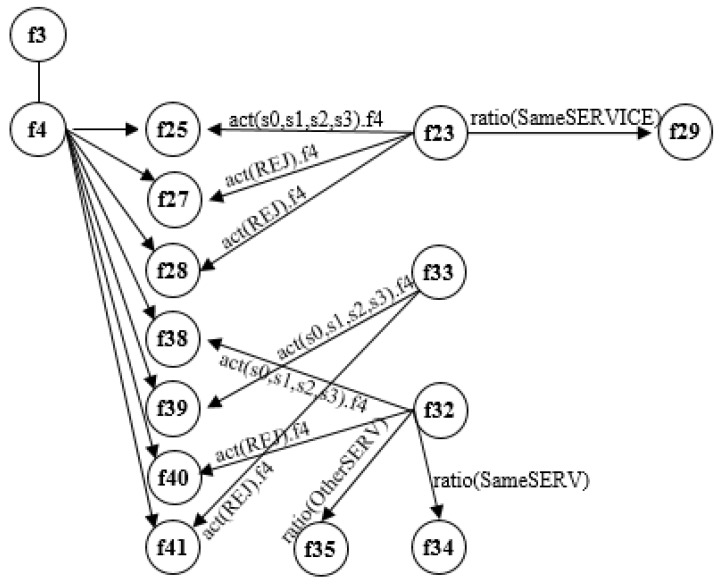
Correlation of each feature.

The main dataset named *KDDCup* contains 42 features and the *KDDTrain*, which was from *KDDCup* abstracted by 15 features in Table 1. The table shows each categorized feature according to calculation including 2 features and non-calculation including 13 features. In the two features area which does not need the calculation, we can obtain the feature value by capturing the network signals such as a flowing protocol and, at that same time, the two features are working independently. On the other hand, the 14 features which need a calculation have to compute by sharing each value it amongst the features. We designed Figure 5 to show the correlation of each feature. It shows which values are going to be transferred to which features. Basically, *f25, f27, f28, f38, f39, f40,* and *f41* need to use a value in *f4*, and *f23* shares the ‘Sum of connections to the same destination IP address’ with *f29*. *f33* sends ‘Sum of connections to the same destination port number’ to *f39* and to *f41*, *f32* sends the ‘Sum of connections to the same destination IP address’ to *f38, f40, f35* and to *f34*. Table 1 contains all features and all classes to each feature. *f3* defines all network services such as *HTTP*, *FTP*, *SMTP*, *telnet*, and *other services*. Because all users including an attacker also use the normal network services, the analysis of *f3* will be useless. Finally, we can say that to analyze *f4* and *(f25, f27, f28)f23, f4* and *(f39, f41)f33, f4* and *(f38, f40)f32* should be the priority when we try to detect anomaly signals in a network.

## 5. Discussion

Network anomaly signal discovery should be real-time. Therefore, real-time data should be continuously applied to anomaly signal analysis results (*a dataset* that continues to be updated) to discover anomaly signals. For real time analysis, it must utilize features that can be immediately found in network signals without prior processing. In this study, we first determine *service(f3)* or *flag(f4)* as potential features. As a result, *flag(f4)* (99.120%) was chosen over *service(f3)* (72.564%) as the basis feature for anomaly signal discovery (Table 2). Refer to Table 2 for further information on the experimental results, Table 4 for the class types defined by *flag(f4)* and the table for compilation of the results of the experiments.

**Figure 6:**
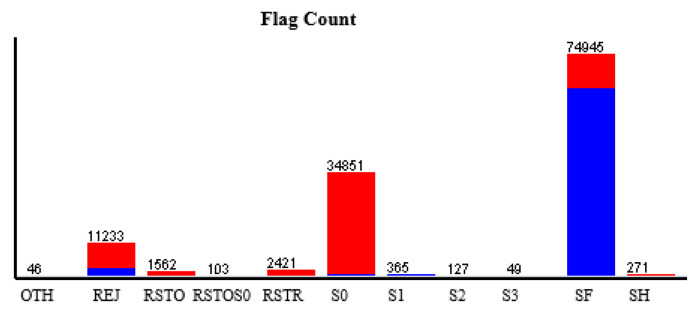
Analysis between service and flag.

For real-time anomaly signal detection, the system must simultaneously upload continuous signal analysis results and determine the basis *feature* for the received signal. As mentioned above, *flag(4)* is chosen from the *KDDTrain dataset* used in this paper. The Figure 5 shows the relationship between the 15 features used, and Table 5 summarizes the number of classes in *flag(4)*. The results according to Table 5 are shown in Figure 6. Red stands for “anomaly” and blue stands for normal. The Figure 5 contains 11 classes defined by the *Flag: OTH, REJ, RSTO, RSTOS0, RSTR, S0, S1, S2, S3, SF,* and *SH*. First, *S0* received 34,851 signals, and they were all determined to be an anomaly (red color). Based on Table 4, *S0* determined an anomaly with 99.7% (0.997) accuracy. *REJ* received 11,233 signals with a normal or anomaly judgment accuracy of 98.7% (0.987).

**Figure 7:**
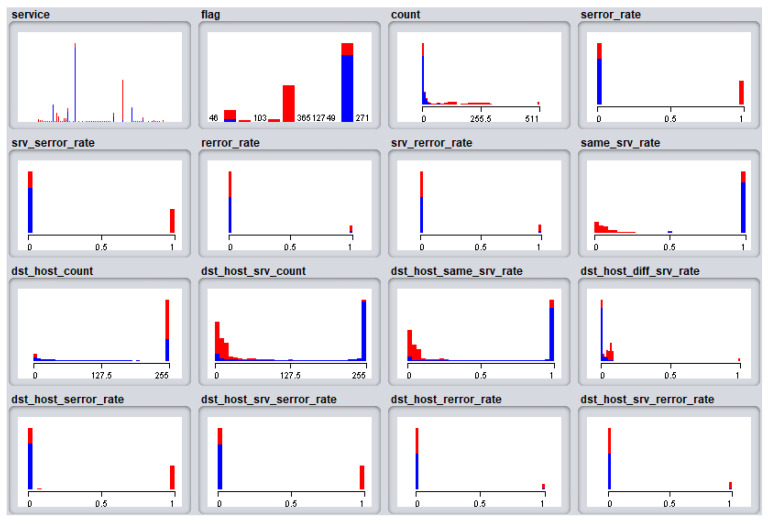
Analysis between features.

The Figure 7 shows the analysis between features, and based on the analysis conducted, the ADM proposed in this work analyzes the received network signals, and concludes that the user responsible for the signal requires closer observation if the class of the flag is *S0* or *REJ*. Furthermore, when reliability is questioned in the analysis by *S0* only, a mixture of *REJ*, *RSTO* and *RSTR* can lead to higher anomaly detection accuracy.

## 6. Conclusions

So far, we have conducted an accuracy analysis based on the feature. The problem with the existing methods has been that real-time processing of the anomaly signal discovery is challenging. To address this problem, we propose an update of the anomaly signal, focused around the features, and a method to detect the anomaly signal based on the selected features that can be obtained from raw data. In this study, the features that can be selected from raw data were *service(f3)* and *flag(f4)*. The *flag(f4)* was selected over *service(f3)* for its relatively higher accuracy score. Nevertheless, the selected feature can be alternated depending on the situation. Since the characteristics of network signals are received in various places, classification based on ML, like conventional methods, is not appropriate for real-time anomaly signal discovery. Therefore, we propose applying the features that can be acquired from raw data to the ADM, so that the model is utilized to preoccupy and monitor signals when features that are deeply associated with abnormal signals are found. In terms of the results, it determined the anomaly with 99.7% (0.997) accuracy in *f(4)(S0)* and in case *f(4)(REJ)* received 11,233 signals with a normal or 171anomaly judgment accuracy of 98.7% (0.987). Future work, it is required for optimal selection of features for detecting more diverse abnormal signals.

## Figures and Tables

**Table 1:** Feature Definition.

Features	Calculation	Classes
*service(f3)*	Do not need	‘aol’, ‘auth’, ‘bgp’, ‘courier’, ‘csnet_ns’, ‘ctf’, ‘daytime’, ‘discard’, ‘domain’, ‘domain_u’, ‘echo’, ‘eco_i’, ‘ecr_i’, ‘efs’, ‘exec’, ‘finger’, ‘ftp’, ‘ftp_data’, ‘gopher’, ‘harvest’, ‘hostnames’, ‘http’, ‘http_2784’, ‘http_443’, ‘http_8001’, ‘imap4’, ‘IRC’, ‘iso_tsap’, ‘klogin’, ‘kshell’, ‘ldap’, ‘link’, ‘login’, ‘mtp’, ‘name’, ‘netbios_dgm’, ‘netbios_ns’, ‘netbios_ssn’, ‘netstat’, ‘nnsp’, ‘nntp’, ‘ntp_u’, ‘other’, ‘pm_dump’, ‘pop_2’, ‘pop_3’, ‘printer’, ‘private’, ‘red_i’, ‘remote_job’, ‘rje’, ‘shell’, ‘smtp’, ‘sql_net’, ‘ssh’, ‘sunrpc’, ‘supdup’, ‘systat’, ‘telnet’, ‘tftp_u’, ‘tim_i’, ‘time’, ‘urh_i’, ‘urp_i’, ‘uucp’, ‘uucp_path’, ‘vmnet’, ‘whois’, ‘X11’, ‘Z39_50’
*flag(f4)*	Do not need	‘OTH’, ‘REJ’, ‘RSTO’, ‘RSTOS0’, ‘RSTR’, ‘S0’, ‘S1’, ‘S2’, ‘S3’, ‘SF’, ‘SH’
*class(f42)*	Do not need	‘normal’, ‘anomaly’
*count(f23)*, *serror_rate(f25)*,		
*rerror_rate(f27)*,		
*srv_error_rate(f28)*,		
*same_srv_rate(f29)*,		
*dst_host_count(f32)*,		
*dst_host_srv_count(f33)*,	Need	
*dst_host_same_srv_rate(f34)*,		
*dst_host_diff_srv_rate(f35)*,		
*dst_host_serror_rate(f38)*,		
*dst_host_srv_serror_rate(f39)*,		
*dst_host_rerror_rate(f40)*,		
*dst_host_srv_error_rate(f41)*		

**Table 2:** Accuracy to the number of features.

Experiment #	Accuracy
42 features,
(+*service(f3)*, +*class(f42)*)	71.093
42 features,
(+*flag(f4)*, +*class(f42)*)	99.564
17 features,
(+*flag(f4)*, +*class(f42)*)	99.120
17 features,
(+*service(f3)*, +*class(f42)*)	72.564

**Table 3:** Flag Code.

Code	Description
*S0*	Connection attempt seen, no reply.
*S1*	Connection established, not terminated.
*SF*	Normal establishment and termination. Note that this is the same symbol as for state S1. You can tell the two apart because for S1 there will not be any byte counts in the summary, while for SF there will be.
*REJ*	Connection attempt rejected.
*S2*	Connection established and close attempt by originator seen (but no reply from responder).
*S3*	Connection established and close attempt by responder seen (but no reply from originator).
*RSTO*	Connection established, originator aborted (sent an RST).
*RSTR*	Established, responder aborted.
*RSTOS0*	Originator sent an SYN followed by an RST, we never saw a SYN-ACK from the responder.
*RSTRH*	Responder sent an SYN ACK followed by an RST, we never saw a SYN from the (purported) originator.
*SH*	Originator sent an SYN followed by an FIN, we never saw a SYN ACK from the responder (hence the connection was “half” open).
*SHR*	Responder sent an SYN ACK followed by an FIN, we never saw an SYN from the originator.
*OTH*	No SYN seen, just midstream traffic (a “partial connection” that was not later closed).

**Table 4:** Detailed accuracy by class with a flag.

TP Rate	FP Rate	Precision	Recall	F-Measure	MCC	ROC Area	PRC Area	Class
0.998	0.006	0.996	0.998	0.997	0.992	0.998	0.998	*SF*
0.997	0.003	0.993	0.997	0.995	0.993	0.999	0.998	*S0*
0.987	0.002	0.981	0.987	0.984	0.983	0.996	0.987	*REJ*
0.945	0.001	0.937	0.945	0.941	0.940	0.992	0.917	*RSTR*
0.943	0.000	0.980	0.943	0.962	0.962	0.981	0.927	*SH*
0.526	0.001	0.674	0.526	0.591	0.594	0.918	0.470	*S1*
0.272	0.000	0.455	0.272	0.340	0.351	0.905	0.218	*S2*

**Table 5:** *flag(f4)* count.

Label	Count
*OTH*	46
*REJ*	11,233
*RSTO*	1562
*RSTOS0*	103
*RSTR*	2421
*S0*	34,851
*S1*	365
*S2*	127
*S3*	49
*SF*	74,945
*SH*	271

## Data Availability

Data sharing not applicable.

## Abbreviations

The following abbreviations are used in this manuscript:

ADM	Anomaly Detection Model
CNN	Convolution Neural Network
ML	Machine Learning
DL	Deep Learning
I/F	Interface
AEWMA	Adaptive Exponentially Weighted Moving Average
EWMA	Exponentially Weighted Moving Average
AS	Anomaly Signal
NS	Normal Signal
